# Obesity and Anterior Abdominal Gunshot Wounds: A Cushion Effect

**DOI:** 10.7759/cureus.19838

**Published:** 2021-11-23

**Authors:** Bharvi Marsha Patel, Alan P Samsonov, Joy R Patel, Elif Onursal, Min-Kyung Jung, Nanette Talty, Gerard A Baltazar

**Affiliations:** 1 Surgery, New York Institute of Technology College of Osteopathic Medicine, Old Westbury, USA; 2 Department of General Surgery, City University of New York, School of Medicine, New York, USA; 3 Department of Anesthesiology, Penn State College of Medicine, Pennsylvania, USA; 4 Department of General Surgery, St. Barnabas Hospital Health System, Bronx, USA; 5 Statistics, New York Institute of Technology College of Osteopathic Medicine, Old Westbury, USA; 6 Surgery, New York University Langone Health/New York University Winthrop Hospital, Mineola, USA

**Keywords:** non-operative management, violence, trauma, obesity, gunshot wound

## Abstract

Background

Although the standard of care for anterior abdominal gunshot wounds (AAGSWs) is immediate laparotomy, these operations are associated with a high rate of negativity and potentially serious complications. Recent data suggest the possibility of selective non-operative management (SNOM) of AAGSWs, but none implicate body mass index (BMI) as a factor in patient selection. Anecdotal experience at our trauma center suggested a protective effect of obesity among patients with AAGSWs, and given the exceptionally high rate of obesity in the Bronx, we sought to analyze the associations of AAGSWs and BMI to inform future trauma research and management. In this study, we aimed to evaluate whether BMI is associated with injury severity, resource utilization, and clinical outcomes of AAGSWs.

Methodology

From our prospectively accrued trauma registry, we retrospectively abstracted all patients greater than 16 years old with Current Procedural Terminology codes associated with gunshot wounds from 2008 to 2016. The electronic medical record was reviewed to define a cohort of patients with at least one AAGSW. Patients were divided into the following cohorts based on BMI: underweight (UW, BMI: <18.5), normal weight (NW, BMI: 18.5-24.9), overweight (OW, BMI: 25-29.9), and obese (OB, BMI: ≥30). Among these cohorts, we analyzed data regarding injury severity, resource utilization, and clinical outcomes.

Results

In this study, none of the patients were UW, 17 (42.5%) patients were NW, 15 (37.5%) patients were OW, and eight (20%) patients were OB. One patient each in the NW and OB cohorts was successfully managed non-operatively, while all others underwent immediate exploratory laparotomy. The mean new injury severity score was significantly lower as BMI increased (NW = 30.9 ± 17.0, OW = 22.9 ± 16.1, and OB = 12.8 ± 13.7; p = 0.039). Patients in the OB cohort were less likely to have abdominal fascial penetration compared to the OW and NW cohorts (p = 0.027 and 0.004, respectively) and sustained fewer mean visceral injuries compared to the OW and NW cohorts (p = 0.027 and 0.045, respectively). OB patients were significantly more likely to have sustained two or more AAGSWs (OB = 27.5%, OW = 6.7%, and NW = 5.9%; p = 0.033), suggesting higher rates of tangential soft tissue injuries. The mean hospital length of stay down-trended as BMI increased but did not achieve statistical significance (NW = 7.4 ± 5.3, OW = 6.6 ± 6.7, and OB = 3.1 ± 2.3; p = 0.19). The OB cohort had the lowest mean hospital charges.

Conclusions

Obesity may yield a protective effect among AAGSW victims, and BMI may provide trauma surgeons another tool to triage patients for SNOM of AAGSWs, potentially diminishing the risks associated with negative laparotomy. Our data serve as the basis for the analysis of a larger patient cohort.

## Introduction

The prevalence of obesity in the United States is increasing, with over 36% of adults qualifying as obese [1,2]. Penetrating abdominal trauma accounts for 35% of patients admitted to urban trauma centers and up to 12% of those admitted to suburban or rural centers. Studies have shown that approximately 40% of firearm homicides and 16% of firearm suicides involve gunshot wounds (GSWs) to the abdomen [3]. Early research suggests a potential mechanical and physiologic benefit of obesity in trauma, including a possible “cushion effect,” “obesity paradox,” and other possible protective factors related to increasing body mass index (BMI) and injury outcome [4-8]. Most studies that examine penetrating trauma and body habitus investigate the association between increasing BMI and abdominal stab wounds [9]. A gap of knowledge exists regarding the relationship between obesity and outcomes of abdominal GSWs.

Although the standard of treatment for anterior abdominal gunshot wounds (AAGSWs) is immediate laparotomy [10], some data suggest that selective non-operative management (SNOM) consisting of clinicians guided by abdominal examination, imaging, and hemodynamic monitoring may also be a successful treatment for AAGSWs [11-14]. Our anecdotal experience based on the institutional practice of immediate laparotomy for AAGSWs noted a high rate of negative and non-therapeutic laparotomies for obese AAGSW patients. The goal of this project is to examine the relationship between BMI and intraoperative findings at laparotomy and clinical outcomes for AAGSWs. We hypothesize that increasing BMI is associated with fewer intraoperative findings at laparotomy and improved outcomes after AAGSW and should be a consideration when selecting patients for non-operative management.

## Materials and methods

After obtaining institutional review board approval (SBH IRB 2017.94), we performed a retrospective cohort analysis using data from the registry of our level two, urban trauma center. We reviewed the electronic medical records (EMRs) of patients 15 years old or older, with Current Procedural Terminology codes associated with GSWs, during an eight-year period from 2008 to 2016. We included all patients with at least one documented AAGSW. We excluded patients with documented mortality within 24 hours of injury, incomplete dataset for analysis, and GSWs exclusively in locations other than the anterior abdomen.

A total of 286 patient charts were reviewed with procedural terminology codes associated with GSW. Of these, 40 patients met the inclusion criteria and were included in the analysis. These 40 patients were grouped into the following three cohorts based on BMI: normal weight (NW, BMI: 18.5-24.9), overweight (OW, BMI: 25-29.9), and obese (OB, BMI: ≥30); there were no underweight (BMI: <18.5) patients in our study group. Primary outcome variables were the operative findings, clinical outcomes, and resource expenditure among the cohorts. Specific data obtained from the registry and EMR included the New Injury Severity Score (NISS), number of AAGSWs, number of viscera injured (each named organ counted as a single visceral injury), duration of operations, index hospital length of stay (LOS), and hospital charges for index admission.

To describe data, mean and standard deviation were computed for continuous variables and frequency and the proportion in percentage for categorical variables. To compare between groups, analysis of variance was used for continuous variables and chi-square test for categorical variables. Statistical significance was evaluated using a p-value of <0.05. SPSS version 23 (IBM Corp., Armonk, NY, USA) was used to conduct all statistical analyses.

## Results

**Figure 1:**
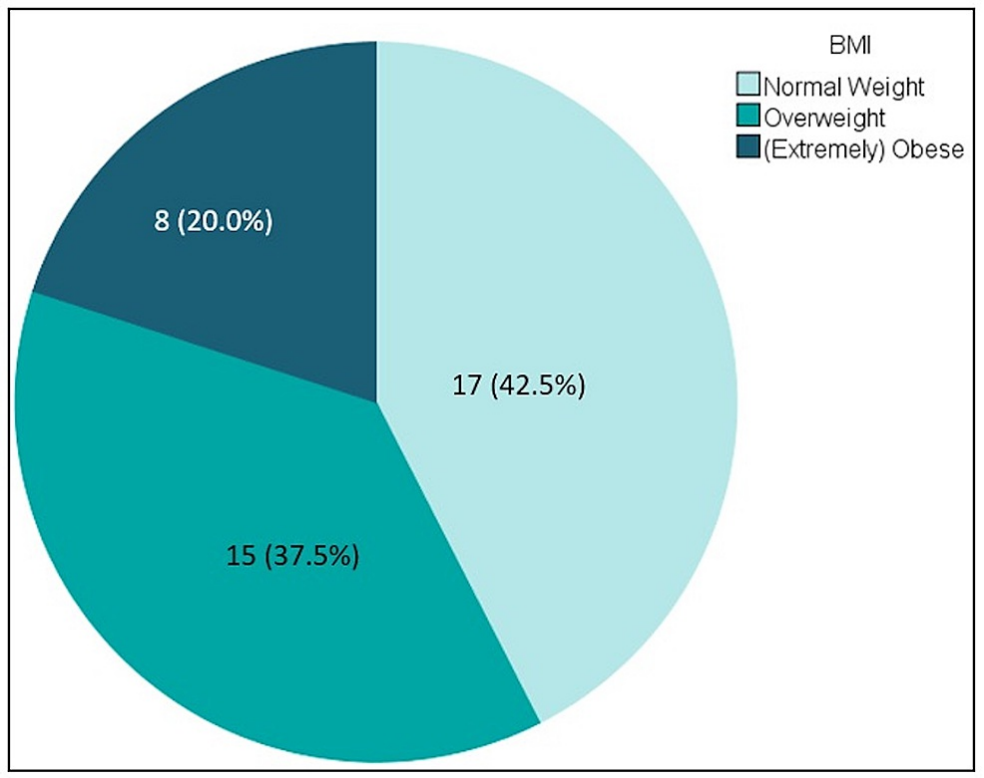
AAGSW patients categorized by BMI. AAGSW: anterior abdominal gunshot wound; BMI: body mass index

A total of 40 patients met the study criteria and were included in the analysis. The number of patients with AAGSWs in each of the three BMI categories is shown in Figure 1.

**Figure 2:**
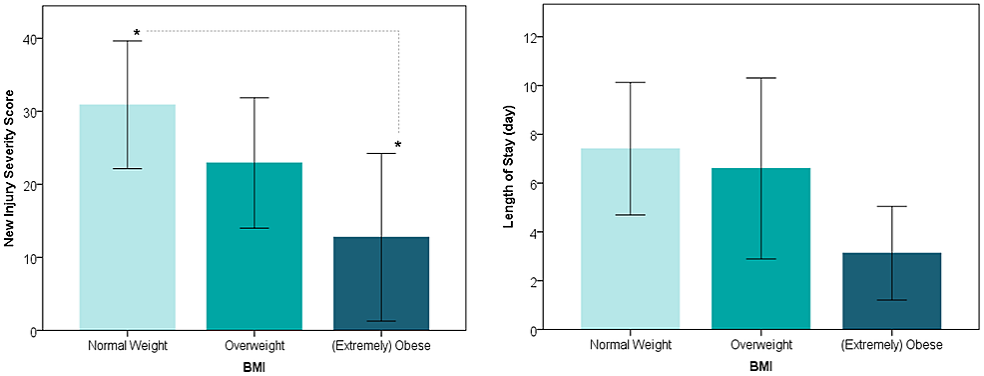
NISS (left) and hospital LOS (right) of AAGSW patients appear to decrease with increasing BMI. *NISS is significantly different between normal weight versus obese cohorts (p = 0.033). NISS: New Injury Severity Score; LOS: length of stay; AAGSW: anterior abdominal gunshot wound; BMI: body mass index

Clinical outcomes are presented in Figure 2. Mean NISS was significantly different among the three cohorts of NW, OW, and OB (p = 0.039). Mean NISS was the highest in cohort NW [30.9 (17.0)], middle in cohort OW [22.9 (16.1)], and the lowest in cohort OB [12.8 (13.7)]. Mean hospital LOS also showed a decreasing trend with increasing BMI, though it did not reach statistical significance (p = 0.19). The average LOS was 7.4 days for cohort NW, 6.6 days for cohort OW, and 3.1 days for cohort OB.

**Figure 3:**
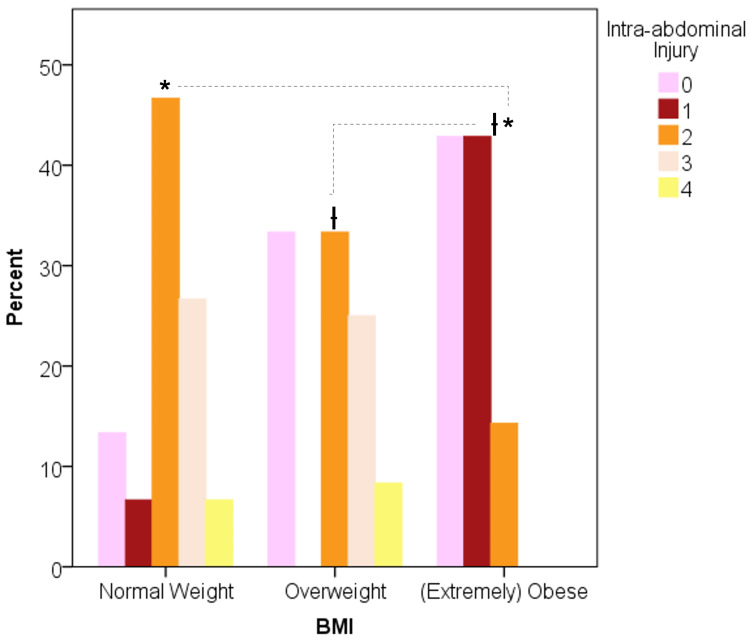
Obese AAGSW patients suffered fewer injured intra-abdominal viscera compared to normal weight (p = 0.004) and overweight (p = 0.027) patients. The number of intra-abdominal injuries is significantly different between *normal weight versus obese (p = 0.004) and between ^ƚ^overweight and obese cohorts (p = 0.027). AAGSW: anterior abdominal gunshot wound; BMI: body mass index

The number of intra-abdominal visceral injuries was significantly associated with BMI groups (p = 0.012), as shown in Figure 3. Two or more intra-abdominal visceral injuries were found in 80% of cohort NW, 66.7% of cohort OW, and 14.3% of cohort OB (Figure 3). Cohort OB had the highest rate (57.1%) of zero visceral injuries compared to cohort OW (16.7%) and cohort NW (13.3%), and therefore the highest rate of non-therapeutic laparotomies.

The number of visceral GSWs was associated with BMI groups. Specifically, 14.3% of cohort OB had two or more visceral injuries, which was the lowest compared to 66.7% of cohort OW (p = 0.027) and 60.0% of cohort NW (p = 0.045).

Mean hospital charges for index admission and mean operative duration was not significantly different among the three cohorts, yet there was a consistent trend that the values were lower in cohort OB than in cohorts OW and NW. Cohort OB had mean total hospital charges of $52,659 and mean operation duration of 83.8 minutes. These values were lower than cohort OW ($85,323 and 135.3 minutes, respectively) and cohort NW ($69,202 and 127.3 minutes, respectively).

The overall hospital LOS decreased with increasing BMI (p = 0.05). The average LOS for obese patients was 3.1 days and was significantly lower than the other two cohorts with LOS of 7.4 days and 6.6 days for normal weight and overweight, respectively.

## Discussion

We present a small but meaningful analysis illustrating the relationship between BMI and AAGSW laparotomy clinical findings and outcomes performed for AAGSWs at a single urban trauma center. Our findings demonstrate that high BMI is significantly associated with lower NISS, shorter hospital LOS, and fewer injured viscera, as well as lower hospital charges and operation duration when compared to lower BMIs. These data correlated with our anecdotal experience of improved outcomes among obese AAGSW victims.

While the ideal measure for central obesity would be the abdominal circumference, this metric was not available for all patients in our study as the setting was the trauma bay. While BMI is a generalized marker for obesity and not specific to any anatomical fat distribution, it is possible that patients with high BMI and average or below-average abdominal circumference may have an increased risk for injury severity than suggested by our study. Ideally, BMI along with abdominal circumference should be evaluated to assess if SNOM is an appropriate treatment approach for any patient.

The positive correlation between increasing BMI and increased protection in the trauma setting may appear counterintuitive because obesity and obesity-associated metabolic syndrome are often responsible for negative clinical outcomes such as superficial adipose infections, anastomotic leak, pneumonia, and septic complications [15]. A potential explanation around the mechanical benefit is that AAGSWs may be less likely to penetrate the peritoneal cavity of obese patients given the typically higher ratio of skin and subcutaneous tissue to intra-abdominal space with increasing BMI [9]; our data appear to corroborate this notion.

Similarly, larger amounts of intra-abdominal fat may increase the likelihood that a bullet’s trajectory will strike fat rather than viscera. Our data suggest that, for AAGSWs, higher BMI is associated with a decreased number of visceral injuries, possibly due to a widening margin of safety that comes with the extra adipose tissue of obese patients. In our study population, obese patients experienced more than one visceral injuries (14.3%) less frequently compared to their overweight (66.7%) and normal weight (80%) counterparts.

A study assessing the severity of motorist crash injury stratified by BMI identified the protective nature of obesity due to the “cushion effect.” An obese habitus was described as “insulation” that could better absorb traumatic force for motorists and be advantageous for patients with higher BMI in blunt trauma [5]. Whether an analogous change in energy transfer and momentum occurs with AAGSWs relative to BMI is unclear and warrants further research. Although our study revealed fewer visceral injuries with increasing BMI, we suspect that this is due to the more tangential nature of GSWs in combination with the change in the amount or distribution of force.

Additionally, the aforementioned “cushion effect” was not only found in blunt trauma among motorists but also in abdominal stab wounds [8]. Although mortality trended upward with increasing BMI, chest and abdomen acute injury severity (AIS) score trended downward [8]. Injury severity scores (ISS) trended downward as well, and the percentage of patients who were more severely injured (ISS ≥ 25) also significantly decreased with increasing BMI. As BMI increased, the rates of peritoneal violation decreased, and the rates of visceral injury and serious injury requiring a therapeutic operation reduced significantly. Of all trauma patients with stab wounds, those in the thinnest group required an operative intervention 3.4 times more frequently than those in the obese group. Among patients with a known peritoneal violation, the thinnest patients also required a surgical intervention twice as more often [9].

Metabolically, increased fat reserves during catabolic states, such as after major trauma, may reduce strain on the immune system and allow more rapid healing and modulated inflammatory response [6]. While we were unable to demonstrate such a relationship at the molecular level in our study, we did observe a downward trend in hospital LOS with an increase in BMI.

The initial management of patients with penetrating trauma was initially explorative laparotomy, but the paradigm and conversation have shifted to include SNOM. A mandatory operative approach for AAGSWs has pitfalls, particularly that up to 21.7% of trauma laparotomies may be negative, and up to 41% of negative laparotomies may result in severe complications [4,14]. The rate of negative laparotomy has been shown to range from 15.7% to 32.5% [4,11], while the rate of non-therapeutic laparotomy after penetrating trauma was 44%, which significantly increased with BMI [9]. This is significant, especially in the context of AAGSWs in patients with increasing BMI, for which we found a direct relationship with more frequent non-therapeutic laparotomies.

The argument for SNOM in managing AAGSWs remains controversial. Proponents for SNOM for GSWs note that, for blunt abdominal trauma and abdominal stab wounds, SNOM is associated with decreased unnecessary laparotomy, reduced LOS, and significant cost savings. Its application should, therefore, be extended to GSWs [13]. Demetriades et al. suggested that 30% of AAGSWs and 67% of posterior abdominal GSWs can be treated successfully with SNOM [13]. Another study confirmed these results by reporting that 86% of penetrating injuries of the chest, 45% of stab wounds to the anterior abdomen, 80% of penetrating injuries to the back, and 25% of AAGSWs can be safely managed conservatively in urban trauma centers with house staff trained in the technique of careful observation for clinical signs of deterioration requiring additional intervention.

Guidelines established by the Eastern Association for the Surgery of Trauma Practice Management Guidelines Committee state that low-resourced trauma centers, especially those with less experience with SNOM, should consider avoiding SNOM, especially for patients with GSWs and close-range stab wounds [12]. Based on data from our low-resourced trauma center, opportunities for SNOM based on BMI may be feasible and should help inform decision-making regarding laparotomy after AAGSW.

Additionally, for low-resourced trauma centers, operation cost is a vital metric to consider in the treatment choice for AAGSW patients. Current data on hospital charges for SNOM is sparse, but Velmahos et al. reported that utilizing SNOM produced significant cost savings in their trauma population [12]. Patients with SNOM had lower charges than those with failed non-operative management ($27,461 ± 29,043 vs. $8,731 ± 9,966; p < 0.001), and their team suggested that had the entire patient cohort undergone SNOM, there would have been a total decreased LOS of 3,560 days and a decrease in hospital charges by $9,555,752 during the eight-year study period, translating to $1,194,469 fewer costs per year [13].

Patient BMI, treatment type, trauma center size, and funding should be considered when managing acute AAGSWs. Our data demonstrate lower hospital charges for AAGSW patients and more frequent non-therapeutic laparotomies with higher BMI. Together, these data suggest the potential for cost savings if SNOM practices inclusive of BMI were adopted for AAGSWs at our low-resourced institution. Further prospective analysis based on such SNOM guidelines is necessary to assess the validity of this concept.

Limitations

Our study has several limitations including the well-described limitations of retrospective, single-center studies. Although showing significant patterns, our study suffers from a very small sample size that decreases the validity of our findings. Moreover, we assessed surrogate measures of body fat and did not use radiologic methods to confirm the degree of adiposity, specifically abdominal adiposity. While current research recommends that waist circumference is the most accurate predictor of obesity-related complications [16], we did not have such data available for our cohort as these patients were seen in a trauma setting. Our study also did not involve any assessment of existing comorbidities in the patients, nor did we ensure that patients in each of the BMI groups were stratified according to common illnesses such as cardiovascular disease or diabetes which may impact their hospital LOS. Lastly, the reduced variance of BMI in our study population may have prevented us from identifying associations that may be observed in populations with greater variance.

## Conclusions

We performed a retrospective cohort analysis of AAGSW patients and noted an association between improved clinical outcomes, shorter hospital stays, and increased BMI. Among AAGSW victims, increasing BMI may be protective against injury severity, peritoneal penetration, intra-abdominal injury, and suboptimal outcomes. BMI may provide trauma surgeons another tool to triage patients with AAGSWs for immediate laparotomy versus SNOM, potentially diminishing the risk of complications associated with unnecessary laparotomy. Our preliminary findings warrant further, multicenter studies to better understand the relationship between obesity and its protective role in clinical outcomes in GSWs.
